# A novel dataset and deep learning object detection benchmark for grapevine pest surveillance

**DOI:** 10.3389/fpls.2024.1485216

**Published:** 2024-12-12

**Authors:** Giorgio Checola, Paolo Sonego, Roberto Zorer, Valerio Mazzoni, Franca Ghidoni, Alberto Gelmetti, Pietro Franceschi

**Affiliations:** ^1^ Research and Innovation Centre, Fondazione Edmund Mach, San Michele all’Adige, TN, Italy; ^2^ Technology Transfer Centre, Fondazione Edmund Mach, San Michele all’Adige, TN, Italy

**Keywords:** *Scaphoideus titanus*, insect detection, yellow sticky traps, deep learning, machine vision, precision agriculture

## Abstract

Flavescence dorée (FD) poses a significant threat to grapevine health, with the American grapevine leafhopper, *Scaphoideus titanus*, serving as the primary vector. FD is responsible for yield losses and high production costs due to mandatory insecticide treatments, infected plant uprooting, and replanting. Another potential FD vector is the mosaic leafhopper, *Orientus ishidae*, commonly found in agroecosystems. The current monitoring approach, which involves periodic human identification of yellow sticky traps, is labor-intensive and time-consuming. Therefore, there is a compelling need to develop an automatic pest detection system leveraging recent advances in computer vision and deep learning techniques. However, progress in developing such a system has been hindered by the lack of effective datasets for training. To fill this gap, our study contributes a fully annotated dataset of *S. titanus* and *O. ishidae* from yellow sticky traps, which includes more than 600 images, with approximately 1500 identifications per class. Assisted by entomologists, we performed the annotation process, trained, and compared the performance of two state-of-the-art object detection algorithms: YOLOv8 and Faster R-CNN. Pre-processing, including automatic cropping to eliminate irrelevant background information and image enhancements to improve the overall quality of the dataset, was employed. Additionally, we tested the impact of altering image resolution and data augmentation, while also addressing potential issues related to class detection. The results, evaluated through 10-fold cross validation, revealed promising detection accuracy, with YOLOv8 achieving an mAP@0.5 of 92%, and an F1-score above 90%, with an mAP@[0.5:0.95] of 66%. Meanwhile, Faster R-CNN reached an mAP@0.5 and mAP@[0.5:0.95] of 86% and 55%, respectively. This outcome offers encouraging prospects for developing more effective management strategies in the fight against Flavescence dorée.

## 1 Introduction

**Figure 1:**
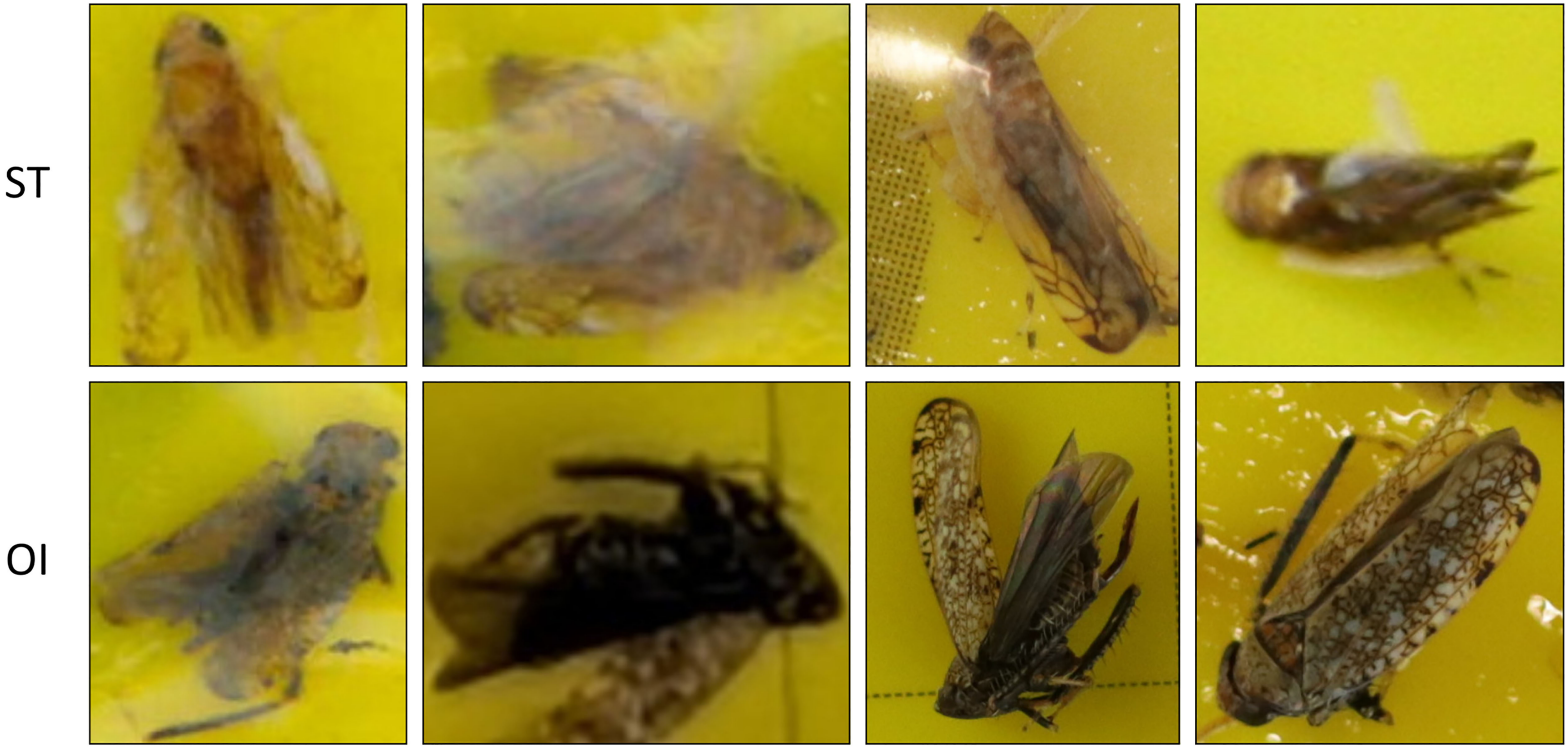
Samples of insect vectors captured by yellow sticky traps. The first row represents examples of the ST class, while the second row shows examples of the OI class.

Among grapevine adversities, Flavescence dorée (FD) is the most severe phytoplasma disease in Europe and for this reason is subject to quarantine measures across the European Union (EFSA et al., 2020). In response to the worsening impact and damages caused by this harmful phytoplasma, the Italian Ministry of Agriculture, Food Sovereignty, and Forests redefined emergency phytosanitary measures in 2023, issuing the Order No. 22/06/2023, No. 4 (G.U. 12/08/2023, n. 188). First discovered in Italy during the early 1970s, it has since spread to most viticultural regions, with epidemic episodes peaking at the end of the century (Morone et al., 2007). FD infection results from the interaction between a phytoplasma and an insect vector, primarily *Scaphoideus titanus* (ST), the American grapevine leafhopper, which is monophagous on *Vitis* plants (Lessio et al., 2014). Nymphs appear in May, while adults emerge at the beginning of July. Both nymphs and adults can acquire the phytoplasma while feeding on infected plants. Once infected, they remain carriers for the rest of their lives, transmitting the pathogen from one grapevine to another (Gonella et al., 2024). Due to its small size, ranging from 4.8 to 5.8 mm, identifying ST without adequate magnification is challenging even for entomologists. Another emerging vector is *Orientus ishidae* (OI), also known as the mosaic leafhopper due to the characteristic color pattern of its wings (**Figure 1**) (Gaffuri et al., 2011; Lessio et al., 2019). Despite the lower transmission efficiency compared to *S. titanus* (Lessio et al., 2016), its widespread presence also in other agroecosystems, such as apple orchards (Dalmaso et al., 2023), make it a potential concern.

The current management strategy for controlling FD involves limiting the spread of the vector through the timely application of insecticides, primarily targeting juveniles, and uprooting the affected plants to prevent the disease from spreading. However, since it has been discovered that adults can also acquire and transmit FD very efficiently, monitoring the dynamics of the ST population has become fundamentally important to determine whether a summer insecticide treatment is necessary (Alma et al., 2018, p. 201). Surveillance of *S. titanus* and *O. ishidae* adults rely on sticky card traps (Pavan et al., 2021), which are left in the vineyard for 7-15 days and then manually checked for the presence of vectors by expert operators. Following trap collection, insect identification is performed in the laboratory by using a stereoscopic microscope. This approach, albeit reliable, is time consuming and represents a bottleneck towards the development of large scale real-time monitoring.

In recent years, technology advancements in FD management have involved two main research avenues (Lee and Tardaguila, 2023):

1. The automated monitoring of vector spread using machine vision techniques on insect traps: such solutions could significantly enhance disease control by enabling real-time mapping and generating large datasets of digitized trap images, allowing for retrospective investigations into the spread of other potential vectors. Two researchers (Ding and Taylor, 2016) pioneered the use of convolutional neural networks (CNNs) for detecting moths from trap images. Subsequently, several studies have applied similar algorithms to yellow sticky traps for various purposes, including monitoring vine pests (Bessa, 2021; Gonçalves et al., 2022), detecting other species of insects, such as Scirtothrips dorsalis (Niyigena et al., 2023) and the European cherry fruit fly (Salamut et al., 2023). Moreover, significant research efforts have been dedicated to developing easily deployable trap systems for real-time detection (Bjerge et al., 2021, 2022; Le et al., 2021; Suto, 2022; Bjerge et al., 2023; Sittinger et al., 2023). Some manufacturers have made available new smart traps for the detection of ST adults, e.g., Trapview (https://trapview.com/) and iSCOUT^®^ COLOR TRAP by Metos (https://metos.global/en/iscout/). Finally, other studies have leveraged open datasets, such as iNaturalist, to benchmark state-of-the-art models for multi-species detection (Ahmad et al., 2022; Kumar et al., 2023; Wang et al., 2023), but these solutions were not specifically developed for research grade applications.
2. Vineyard monitoring using imaging techniques on symptomatic plants: computer vision combined with multispectral and hyperspectral imaging or remote sensing has shown promising results for early detection of grapevine diseases (Silva et al., 2022; Tardif et al., 2022, 2023).

The focus of this study concerns the first line of research, specifically FD vectors captured by sticky card traps. In this area, the lack of high-quality datasets necessary for model training represents the major limitation to the development of automatic monitoring solutions of ST. In this paper, we address this gap by presenting and making available a fully annotated dataset of yellow sticky trap images, with insect identifications carried out by a team of expert entomologists. The dataset has to be intended as a reference source for establishing an autonomous and accurate pest identification system against the FD spread. In addition, we demonstrate its potential use by benchmarking two state-of-the-art object detection architectures with different image processing techniques.

## 2 Materials and methods

### 2.1 Data collection

The efficiency of a deep learning model depends on the quality and quantity of data used for the training. Considering the scope of our investigation, we focused on yellow sticky traps (YST) (Glutor, Biogard^®^, 10x25 cm), positioned in vineyards from different sites in Trentino (northern Italy) from July to November 2023, when ST adults occur. YST were exposed for a maximum of 14 days.

**Figure 2:**
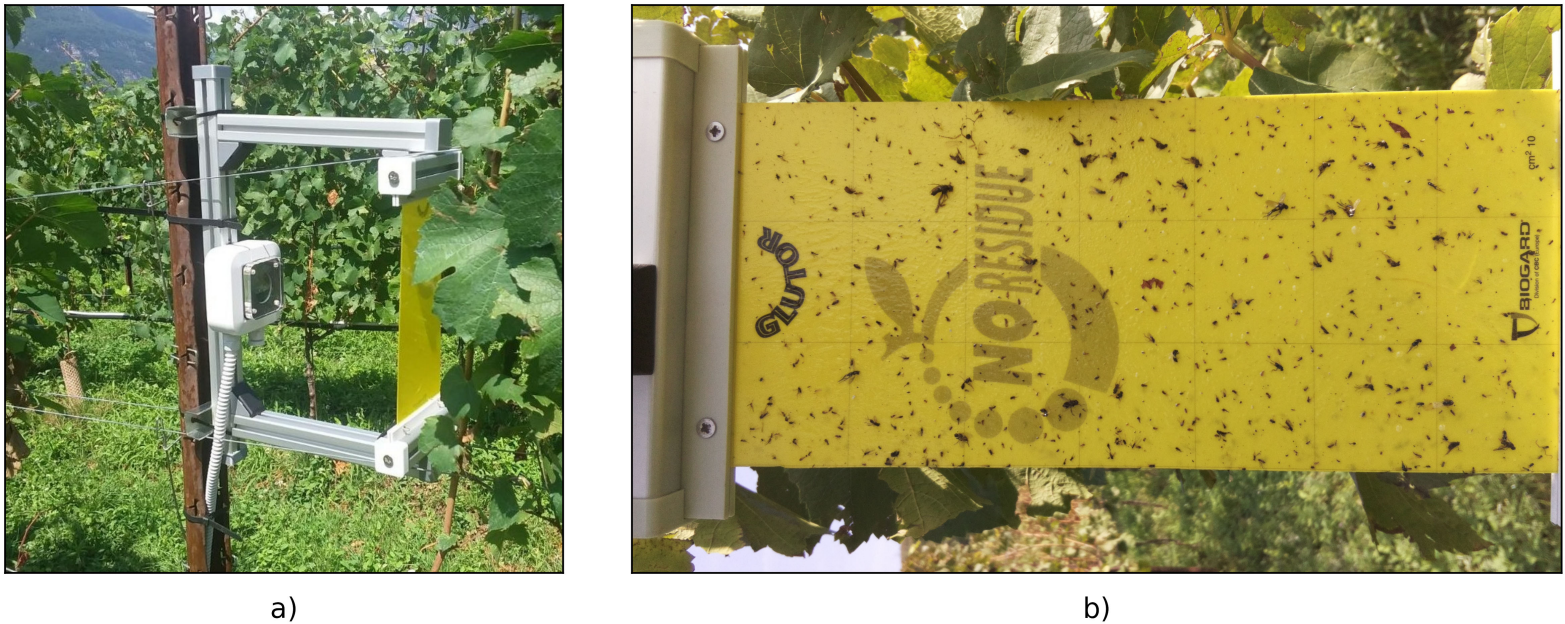
Smart-trap prototype **(A)** and its camera framing **(B)**.

Due to the practical challenges in sample collection, images were obtained through four distinct methods (**Table 1**):

- photos taken directly in the field;
- images of stored YST (T = 5 ± 1°C), and deceased reared insects on empty traps within a controlled greenhouse environment;
- digital scans of YST collected during regular monitoring activities in the fields;
- photos from a smart trap prototype installed in our experimental vineyard (**Figure 2**).

**Table 1:** Structure of the dataset, showing the number of images from each data source and the corresponding class annotations.

Image source	Number of images	ST annotations	OI annotations	Number of background images
Field	18	3	101	8
Laboratory	157	473	863	8
scanned	390	853	542	84
smart-trap	50	0	0	50

Digitally scanned YST made the primary contribution to our dataset since this method of acquisition allowed us to avoid common camera issues related to external interference, such as focusing and lighting, while also maximizing resolution. However, it presented drawbacks such as suboptimal visual conditions due to the risk of insect squeezing during the scanning process and possible reflections of nylon bags in which yellow traps are stored.

Regarding the smart trap, the device consists of several commercial components mounted on a customized printed circuit board (PCB). Specifically, the diurnal 3-hourly (9:00 AM, 12:00 AM, 3:00 PM, 6:00 PM) time-lapse images have been captured by the 8 MPixel Raspberry Pi camera module V2 (Raspberry Pi Foundation, Cambridge, UK) connected to the Raspberry Pi zero W single board computer. Each image consists of 3280 × 2462 pixels and the final size of the jpg file is about 5 MBytes. A Witty Pi 3 mini clock and power management board controlled the ON/OFF scheduled sequence. Images were sent back to the server via WiFi, by means of a Secure Copy Protocol (SCP) file transfer protocol. These images were repurposed as background images given the absence of target insects due to mandatory treatments against the spread of FD.

The final dataset consists of 615 images which also include the images of 150 traps where the two target insects were not detected. These were included to add variety so that the network can properly learn to distinguish the target objects from other insects. Insect annotations comprise 1329 ST and 1506 for OI, ensuring an almost class-balanced dataset.

### 2.2 Data pre-processing

**Figure 3:**
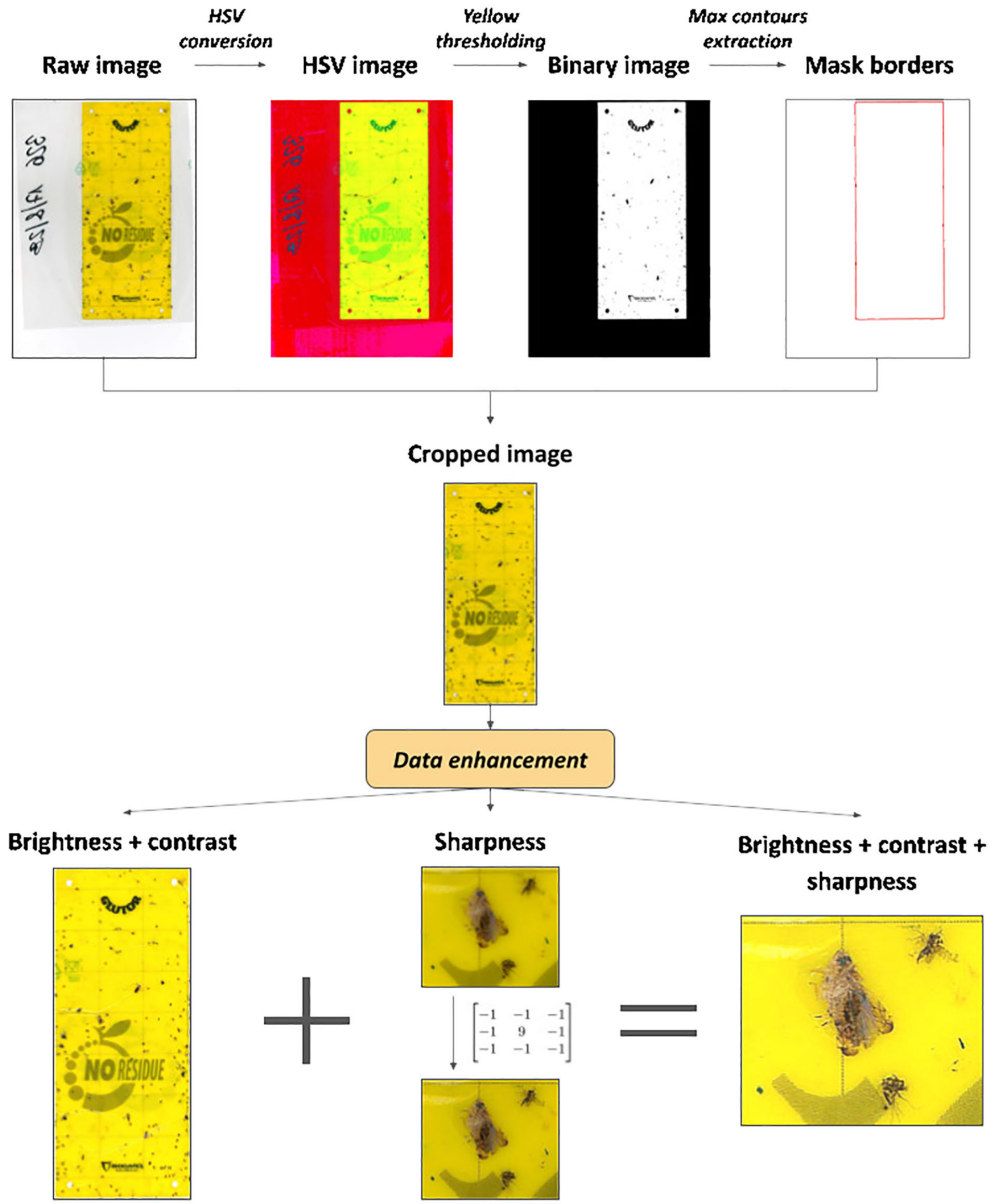
Flowchart diagram of data pre-processing operations.

An automated cropping procedure, inspired by (Bessa, 2021), was implemented using the Python library OpenCV (Bradski, 2000) to remove unnecessary background information outside of the yellow trap. The workflow is outlined in **Figure 3**.

The original images were first converted to the HSV (Hue, Saturation, Value) color space and then segmented by defining two yellow thresholds. Subsequently, the algorithm identified contours in the binary mask image and extracted the largest contour based on its area. Using the coordinates of the bounding rectangle around this contour, the cropping operation on the original images was performed.

Before proceeding with data annotation, enhancement techniques were applied to the images to improve image quality and consequently model performance (Ding and Taylor, 2016; Pang et al., 2022; Suto, 2022). Specifically, brightness, contrast and sharpness parameters were adjusted to increase insect visibility and reduce the impact of lighting variations. Using OpenCV, the addWeighted function modifies brightness and contrast by calculating the weighted sum of two arrays as: α⋅image+β⋅image+γ . We set α=1.1 , β=10 and γ=0 to meet visual requirements. Additionally, a sharpening filter was applied using the filter2D function to enhance image details.

### 2.3 Object detection models

Object detection tasks perform both localization and class recognition, allowing to identify multiple objects in a single image. These algorithms work by drawing bounding boxes around object targets along with a confidence score, indicating the likelihood that the bounding box contains the object.

Currently, object detection models consist of Convolutional Neural Networks (CNNs) (Krizhevsky et al., 2012), which are typically composed of three main components: the backbone network, the neck, and the head. The backbone, commonly a pre-trained CNN, extracts and encodes features from the input data; the neck further processes these features, enhancing their representational and informative power. One example is Feature Pyramid Network (FPN) (Lin et al., 2019). Finally, the head predicts the bounding boxes and class probabilities of detected objects based on the previously extracted information.

Object detectors can be categorized into two main types depending on their architecture: one-stage and two-stage detectors. The former predicts bounding boxes and class probabilities in a single forward pass, while the latter, as the Region-based Convolutional Neural Networks (Girshick et al., 2014) first proposes regions of interests (ROIs) in the image and then predicts the class and refine the bounding box for each proposed region. In our study, we chose to use the latest state-of-the-art detection architectures: YOLOv8 and Faster R-CNN. We selected these algorithms based on their respective strengths and suitability for our specific requirements.

For data annotation, we employed the open-source software CVAT (CVAT.ai Corporation, 2023). Under the guidance of entomologists, we labeled all instances related to the target pests even though their visual appearance could sometimes confuse the detector and introduce noise. Annotations were exported in YOLO format, which consists of string lines written as:

(class_id x_box_centre y_box_centre width height)

#### 2.3.1 YOLO

YOLO (You Only Look Once) is a popular family of one-stage object detection models known for their speed and efficiency (Redmon et al., 2016). Unlike two-stage methods, YOLO solves detection as a regression problem.

Developed by Ultralytics (Glenn Jocher et al., 2023) and released in January 2023, YOLOv8 serves as the latest advancement in the YOLO family (as of the time of writing). It incorporates several improvements, including mosaic data augmentation, anchor-free detection, a more powerful backbone network, a decoupled head, and a modified loss function. Among the various model variants, we focused on YOLOv8s due to our computational constraints.

#### 2.3.2 Faster R-CNN

Faster R-CNN implements Region Proposal Network (RPN) for generating potential bounding box proposals and a bounding box regression and classification network for refining these proposals and predicting the class labels (Ren et al., 2016). We implemented the algorithm using the Detectron2 framework (Wu et al., 2019), a cutting-edge tool developed by Facebook for a wide range of computer vision tasks.

For our study, we used the faster_rcnn_R_50_FPN_3x.yaml configuration, which uses a ResNet-50 (He et al., 2016) backbone network and integrates the FPN network to generate multiple feature maps of different scales. This configuration provides a good balance between speed and accuracy. The “3x” designation refers to the length of the training schedule (He et al., 2018).

### 2.4 Experiments and evaluation

#### 2.4.1 Experiment design

We conducted several tests to benchmark the machine vision models, assessing the impact of the different preprocessing steps on their detection capability. Initially, we evaluated the effect of image enhancements to understand how it influenced training performance. The second test aimed to assess the impact of image resolution, as it is indeed known to significantly affect performance, albeit with a considerable increase in computational cost. In our tests, we focused on 640 and 1280 pixel images, both considered reasonable sizes to balance computational time and performance, while avoiding memory constraints. The algorithm automatically resized the images, setting their longest dimension to the chosen value, while preserving the original aspect ratio.

A similar test was conducted to evaluate the YOLOv8 built-in data augmentation. Based on several hyperparameters, default transformations are randomly applied to the training data to increase the diversity and size of the dataset. We conducted two training runs to compare the effects of default hyperparameters with their zeroing (see **Supplementary Table 1**).

From a more fundamental perspective, we explored whether, for our dataset, a deep learning model learns better when trained on one class (one insect species) at a time compared to binary-class detection. To get more insight on this aspect, we conducted an additional test by considering both classes as a single entity, labeled “pest”.

Finally, we evaluated the model architectures. After implementing Faster R-CNN with three different augmentation settings, we compared the best configuration with the one-stage detector, YOLOv8s.

To ensure a more robust estimate of model performance and to allow an honest estimation of the variability of the prediction metrics, we implemented a 10-fold Cross Validation scheme (Hastie et al., 2009). Given the challenges associated with class stratification in object detection tasks, we selected a random K-fold splitting that achieved an acceptable balance in the distribution of the two classes (**Supplementary Table 2**).

#### 2.4.2 Evaluation metrics

To assess the performance of an object detection model, we examine its ability to correctly identify the object’s class and accurately predict their bounding box coordinates. Each prediction is characterized by a value of Intersection over Union (IoU) and confidence score. IoU, based on the Jaccard index, evaluates the degree of overlap between the predicted bounding box and the ground truth. Values range between 0 to 1, where a value closer to 1 implies a better alignment between the predicted and ground truth bounding boxes. The confidence score, instead, indicates the likelihood that the object in the bounding box actually belongs to a specific category. Based on these values, correct predictions are classified as True Positives (TP), while False Positives (FP) include detections of nonexistent target objects, which in our case are insects wrongly identified as ST or OI or misplaced detection of existing objects. False Negatives (FN) encompass all unpredicted ground truth bounding boxes. It’s worth noting that True Negatives (TN) are not considered in object detection, as there exists an infinite number of bounding boxes that should not be detected within an image (Padilla et al., 2020).

From these statistics, we can derive several performance indices, including Precision and Recall. Precision (Equation 1) measures the model’s ability to identify true objects while minimizing the number of incorrect annotations. Conversely, Recall (Equation 2) focuses on the model’s ability to identify all correct objects (TP), regardless of incorrect annotations. Ideally, a perfect model would have both high Precision and high Recall. For insect detection, we opted for low values of confidence score to make the model generate more predictions. This approach results in higher Recall, minimizing FN at the expense of increasing FP (Wenkel et al., 2021).

Lastly, F1-score (Equation 3) shows the harmonic mean of Precision and Recall, considering both FP and FN.


(1) $$Precision=\frac{TP}{TP+FP}$$

(2) $$Recall=\frac{TP}{TP+FN}$$

(3) $$F1\;score=\frac{2}{precision^{-1}+recall^{-1}}$$

IoU and precision-recall measures are used to compute Average Precision (AP) (Equation 4) for each class. By leveraging the area under the precision-recall curve (AUC-PR) and different thresholds of IoU, AP was first estimated using the 11-point interpolation method in the VOC2007 challenge (Everingham et al., 2015) to reduce the zig-zag behavior of the curve. The most common IoU values are 0.5 and 0.75, corresponding to AP@0.5 and AP@0.75, respectively, while AP@[0.5:0.95] represents instead the average precision across ten IoU thresholds varying from 0.5 to 0.95 with a step size of 0.05. Mean Average Precision (mAP) (Equation 5) is then calculated as the mean over all classes, serving as the benchmark metric to evaluate object detection model performance.


(4) $$AP@\alpha =\int_{0}^{1}p\left(r\right)dr$$

(5) $$mAP@\alpha =\frac{1}{n}\sum_{i=1}^{n}AP_{i}for\;n\;classes$$

#### 2.4.3 Experimental setup

Training, validation and inference tests were executed on Amazon Web Services (AWS) virtual machines using a g5.2xlarge instance, which belongs to the GPU instance family. It is equipped with 8 vCPUs, 32.0 GiB of memory, and a NVIDIA A10G with 24.0 GiB of video memory. The configuration settings for each experiment, partially tuned to comply with hardware constraints, are saved in the corresponding YAML files, which are provided in the **Supplementary Material**.

## 3 Results

**Figure 4:**
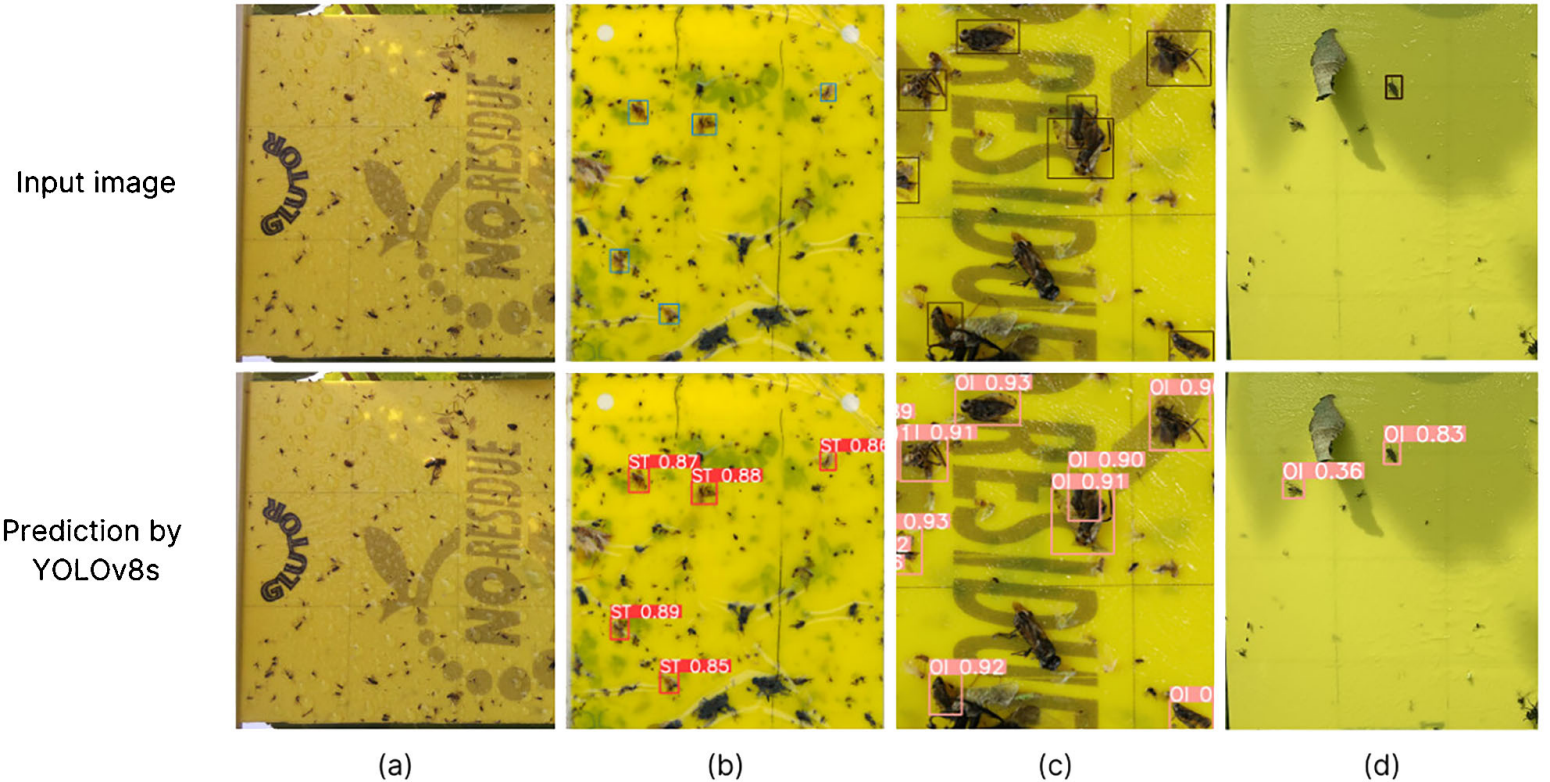
Examples of zoomed insect images with predicted bounding boxes. Red and pink colors represent respectively the detections of ST and OI classes. **(A)** photos from the smart trap; **(B)** details from scanned trap images; **(C)** photos in the laboratory; **(D)** pictures from the field.

**Figure 4** displays examples of model predictions, featuring randomly selected zoomed-in images from the four different sources, along with both ground-truth and predicted annotations. Bounding boxes, obtained from one of the YOLOv8s model tests, are displayed with specific class colors and their corresponding confidence scores. These photos provide a clear view of the model’s performance across various scenarios. For instance, in the scanned trap image (**Figure 4B**), all insects are detected accurately. Challenges arise in **Figure 4C**, where the photo presents a dense concentration of OI bounding boxes, making accurate detection more difficult. Similarly, in **Figure 4D**, the greater distance and ambient light conditions contribute to an increase in both FN and FP.

Quantitative results are presented following the experimental workflow, starting with the YOLO algorithm and moving to Faster R-CNN. Performance metrics are expressed as the mean and standard deviation across the 10 folds (**Table 2**), highlighting the variability of cross-validation splits.

### 3.1 Performance of YOLO models

**Figure 5:**
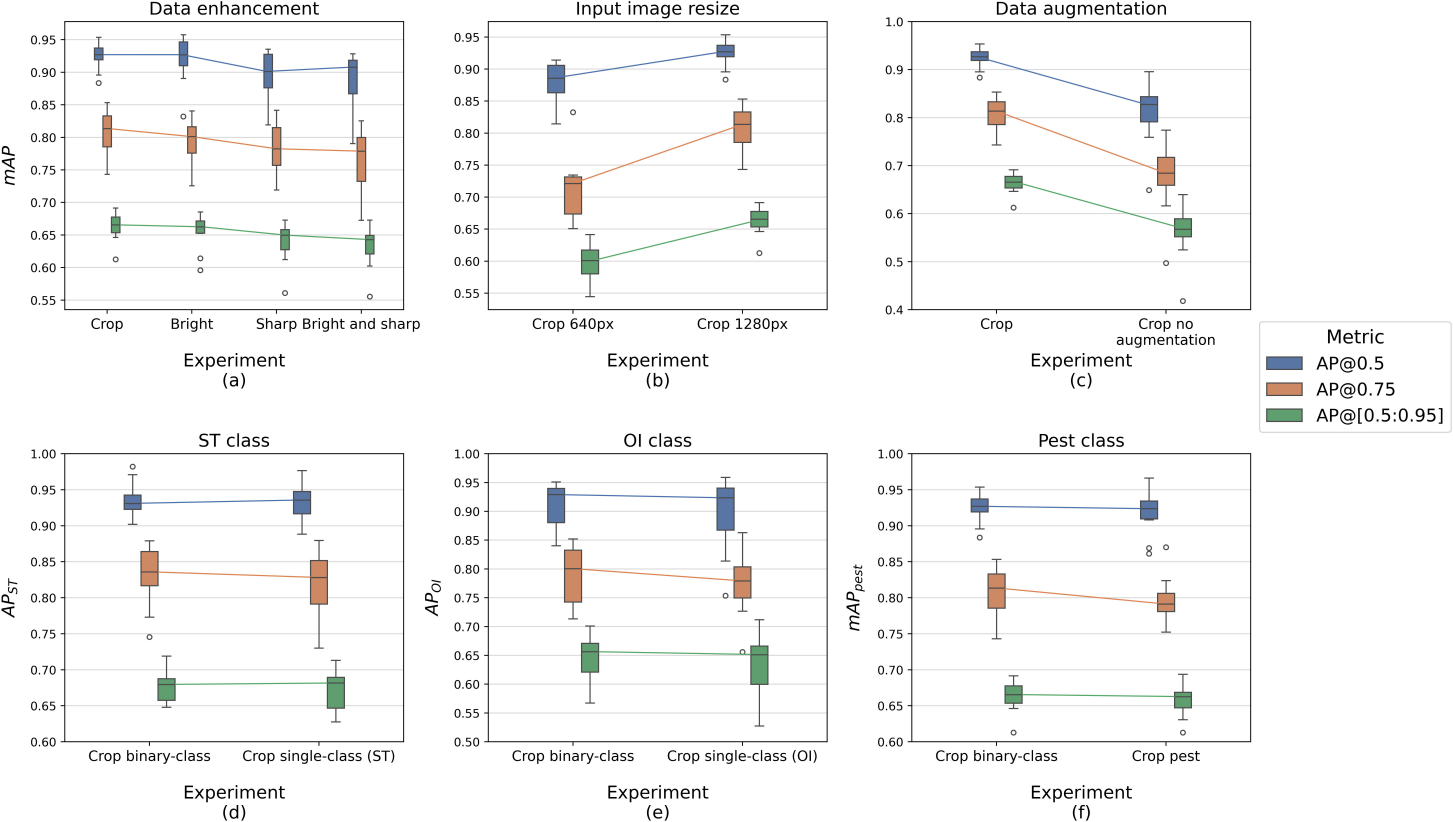
mAP evaluation of YOLO experiments: **(A–C)** represent the comparison on input image modifications; **(D–F)** include the class-oriented tests.

**Figure 5** summarizes the YOLO experiments on input image modifications and class detection, comparing the three Mean Average Precision (mAP) discussed in section 2.4.2, mAP@0.5, mAP@0.75, mAP@[0.5:0.95]. The **Supplementary Material** includes the corresponding Precision-Recall curves obtained during validation at the specific confidence thresholds.

From the comparison of data enhancements (**Figure 5A**), we observe a clear similarity between the Crop and Bright models. Results show mAP@0.5 values ranging from 0.9 to 0.95, mAP@0.75 above 0.8, and mAP@[0.5:0.95] between 0.65 and 0.7, with a difference of less than 2% in the other metrics (see **Table 2**). On the other hand, Sharp and Bright_and_sharp models achieved slightly lower results and higher variabilities, with all mAP values dropping by up to 3%, especially the latter.

**Table 2:** Insect detection performance of the 8 tests conducted.

Index	Test	Configuration	mAP@0.5	mAP@0.75	mAP@0.5:0.95	Precision	Recall	F1 score
**1**	**Data enhancement**	Crop	0.92 ± 0.02	0.81 ± 0.03	0.66 ± 0.02	0.89 ± 0.05	0.89 ± 0.03	0.89 ± 0.03
		Bright	0.92 ± 0.04	0.79 ± 0.04	0.66 ± 0.03	0.90 ± 0.03	0.87 ± 0.06	0.89 ± 0.04
		Sharp	0.90 ± 0.04	0.78 ± 0.04	0.64 ± 0.03	0.89 ± 0.04	0.85 ± 0.04	0.87 ± 0.04
		Bright and sharp	0.89 ± 0.04	0.77 ± 0.05	0.63 ± 0.03	0.87 ± 0.04	0.83 ± 0.06	0.85 ± 0.04
**2**	**Input image size**	Crop 640	0.88 ± 0.04	0.71 ± 0.05	0.60 ± 0.03	0.87 ± 0.03	0.82 ± 0.05	0.84 ± 0.04
		Crop 1280	0.92 ± 0.02	0.81 ± 0.03	0.66 ± 0.02	0.89 ± 0.05	0.89 ± 0.03	0.89 ± 0.03
**3**	**Data augmentation**	no augmentation	0.81 ± 0.07	0.67 ± 0.08	0.56 ± 0.06	0.86 ± 0.03	0.72 ± 0.12	0.78 ± 0.08
		default augmentation	0.92 ± 0.02	0.81 ± 0.03	0.66 ± 0.02	0.89 ± 0.05	0.89 ± 0.03	0.89 ± 0.03
**4**	**Single class ST**	Binary class*	0.94 ± 0.02	0.83 ± 0.04	0.68 ± 0.03	0.91 ± 0.03	0.90 ± 0.04	0.90 ± 0.03
		Single class	0.93 ± 0.03	0.82 ± 0.05	0.67 ± 0.03	0.92 ± 0.03	0.90 ± 0.04	0.91 ± 0.03
**5**	**Single class OI**	Binary class*	0.91 ± 0.04	0.79 ± 0.05	0.65 ± 0.04	0.88 ± 0.08	0.88 ± 0.06	0.87 ± 0.05
		Single class	0.90 ± 0.07	0.77 ± 0.06	0.64 ± 0.05	0.89 ± 0.08	0.84 ± 0.08	0.86 ± 0.07
**6**	**Mono class pest**	binary class	0.92 ± 0.02	0.81 ± 0.03	0.66 ± 0.02	0.89 ± 0.05	0.89 ± 0.03	0.89 ± 0.03
		single_cls=True	0.92 ± 0.03	0.80 ± 0.03	0.66 ± 0.02	0.90 ± 0.02	0.87 ± 0.05	0.88 ± 0.03
**7**	**Data augmentation in Detectron2**	Default Trainer	0.86 ± 0.06	0.66 ± 0.07	0.55 ± 0.05	–	–	–
		no Flip transformation	0.84 ± 0.07	0.64 ± 0.07	0.53 ± 0.05	–	–	–
		Augmentation	0.83 ± 0.05	0.58 ± 0.07	0.50 ± 0.04	–	–	–
**8**	**Model architecture**	Yolov8s	0.92 ± 0.02	0.81 ± 0.03	0.66 ± 0.02	0.89 ± 0.05	0.89 ± 0.03	0.89 ± 0.03
		Faster R-CNN	0.86 ± 0.06	0.66 ± 0.07	0.55 ± 0.05	–	–	–

The metrics include the mean and standard deviation values between 0 and 1 among the 10 folds. *To compare the test with the single-class training, we show the corresponding Average Precision (AP) value of the binary-class training.

The impact of input image resizing (**Figure 5B**) is more pronounced, particularly in terms of mAP@0.75 and Recall (**Table 2**). This observation suggests that image resolution becomes increasingly critical when higher IoU thresholds are required or for the complete detection of ground-truth annotations.

**Figure 5C** clearly demonstrates the effect of data augmentation during training. Without transformations, the model did not exceed 0.81 in mAP@0.5, 0.67 in mAP@0.75, and 0.57 in mAP@[0.5:0.95], with a low Recall of 72%. Training with data augmentation drastically improves these metrics, particularly mAP@0.75 and Recall (**Table 2**).

Regarding class detection tests, **Figure 5D** shows no significant difference between the Average Precision (AP) of ST for both the binary-class and single-class models. The same holds true of the OI class (**Figure 5E**), with subtle differences of less than 2%, except for a 4% increase in Recall for the binary-class model (**Table 2**). Finally, the last plot compares the mAP of the binary-class model and the AP of the pest-class model, both achieving high results: 0.92 mAP@0.5, 0.8 mAP@0.75, and 0.66 mAP@0.5:0.95, with 90% Precision, 87% Recall, and 88% F1 score as shown in **Table 2**.

### 3.2 Performance of Faster R-CNN models

Faster R-CNN results include an assessment of mAP performance when changing the augmentation settings. Three tests were conducted, named Default, Augmentation, and No_augmentation, each based on specific transformations applied during training, similarly to the YOLOv8 built-in data augmentation.

- Default: **This test used the two default Detectron2 transformations**, ResizeShortestEdge and RandomFlip. **The first resizes the image while keeping the aspect ratio, while the other operation flips the image horizontally or vertically with a given probability;**
- Augmentation: **This test introduced additional Detectron2 transformations, including** RandomBrightness, RandomContrast, RandomSaturation, RandomRotation, RandomLighting, **along with** ResizeShortestEdge **and** RandomFlip. **These transformations randomly alter the intensity of image enhancements during training to augment the diversity of the training data;**
- No_augmentation: **This test represented the default training configuration without applying RandomFlip to input images**.

For further details on the code, please refer to our GitHub repository, https://github.com/checolag/insect-detection-scripts.

**Figure 6:**
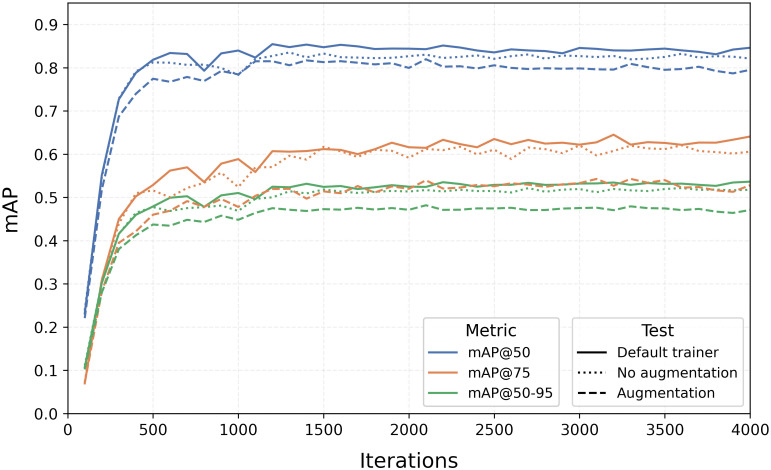
Training curves of mAP@0.5, mAP@0.75, and mAP@[0.5:0.95] for the three augmentation tests with the Faster R-CNN algorithm.

Since Detectron2 does not provide Precision and Recall metrics, we monitored the progress of mAP over iterations for the three tests, as depicted in **Figure 6**. From the graph, we observed two main trends: the default configuration notably outperforms the model with augmentation, and maximum values are generally reached within the first 1500 iterations, after which they remain relatively constant.

As Detectron2 only saves the last model and not the best one, the metric values in **Table 2** were derived considering the iteration at which the model of each split achieved the best results in terms of mAP@0.5, mAP@0.75, and mAP@[0.5:0.95]. Although the differences are small, they are relevant, with the default run reaching 86% in mAP@0.5, 66% in mAP@0.75, and 55% in mAP@[0.5:0.95].

### 3.3 Comparison of the algorithms

This section concludes the experimental evaluation of model architectures, highlighting the difference between the optimal configurations of Faster R-CNN and YOLOv8 that can be computationally managed by our hardware system. Specifically, we compare the chosen Faster R-CNN version with default augmentation settings against YOLOv8 with an input size of 1280 pixels and default augmentation hyperparameters. Both models were trained using only cropped input images.

**Figure 7:**
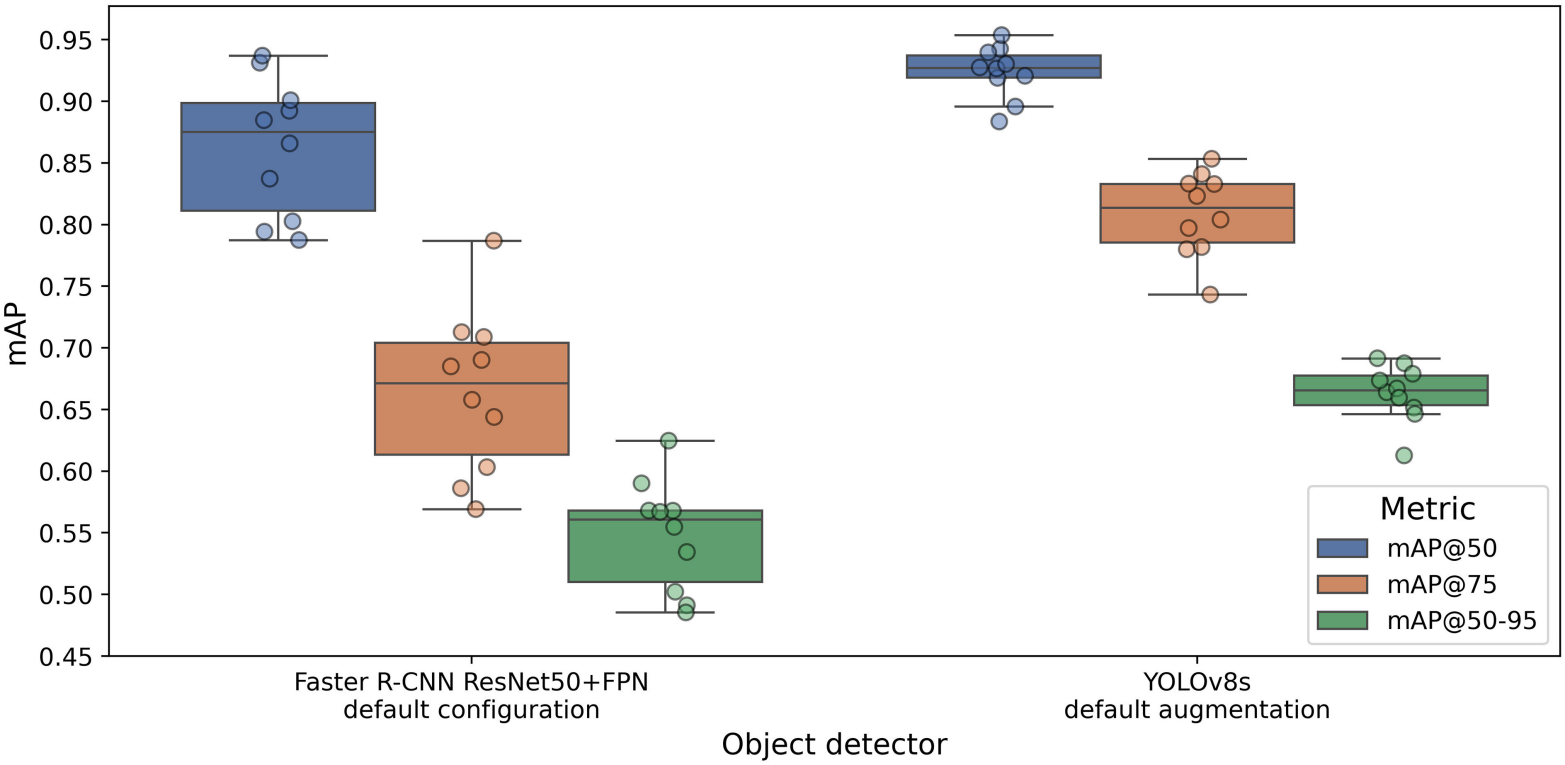
Comparison of mAP@0.5, mAP@0.75, and mAP@[0.5:0.95] values between the optimal configuration of Faster R-CNN and YOLOv8s.

The boxplots in **Figure 7** illustrate mAP@0.5, mAP@0.75, and mAP@[0.5:0.95] across the 10 folds of cross-validation. We observe how YOLOv8s outperforms Faster R-CNN in terms of both accuracy and robustness. The percentage difference between the average values exceeds 6% in mAP@0.5, 15% in mAP@0.75, and more than 10% in mAP@[0.50:0.95]. Moreover, the size of the boxplot clearly shows the higher prediction variability of Faster R-CNN compared to YOLOv8. As shown in **Table 2**, the performance of the two-stage algorithm is highly dependent on the validation split, with a standard deviation of about 6%, compared to YOLOv8’s 2%.

## 4 Discussion

Building on the previously mentioned work on ST detection (Bessa, 2021), which our study aims to expand, this benchmark has demonstrated the effectiveness of object detection algorithms in recognizing ST and OI on yellow sticky traps. We showed how a standardized acquisition procedure, – particularly in the scanned images – combined with a color segmentation, can achieve strong detection performance. Automated detection of FD vectors has proven both feasible and effective, supporting essential pest management strategies against the spread of this grapevine disease (Lee and Tardaguila, 2023).

Interestingly, the first test revealed that enhancing sharpness did not improve the model performance. This modification appeared to introduce noise to the image, which the model interpreted as irrelevant information. Conversely, variations in brightness and contrast resulted in similar detection accuracy as the non-processed dataset, suggesting that the original dataset was already suitable for training, and additional changes did not provide any further benefits. Further studies should be conducted to understand the relationship between model architecture and image processing, with the aim of optimizing the model training process. The use of higher resolution images significantly improved mAP values, with more pronounced effects observed at higher IoU values. However, in case of limited computational resources, an image size of 640 pixels proved to be a good compromise between accuracy and computing time. In accordance with a similar study (Dang et al., 2023), YOLOv8 built-in augmentation resulted in actual improvements, further demonstrating the effectiveness of the default hyperparameter settings.

Class-oriented tests revealed that single-class detectors did not perform better than multi-class models. No significant changes in terms of TP, FP and FN were noted when trained on one class at a time; in fact, they obtained equal or lower results, as seen with the OI class. Even when the classes were combined under a single target label, the differences were minimal. This suggests that binary-class training is a viable strategy for maximizing performance and feedback information. The reason why the ST class achieves higher results and lower variability than OI could be attributed to the distribution of annotations. ST labels are primarily concentrated in one source of image, i.e. the scanned images, which constitutes the majority of the dataset. In contrast, OI annotations were present in all image types, which vary significantly from each other. Some images contain dense clusters of labels, making the detection more challenging.

The Faster R-CNN tests yielded unexpected results. Adding several transformations appeared to confuse the model, resulting in lower mAP values, especially for higher IoU values. While augmentation is typically beneficial, enabling the model to learn under various situations such as different lighting, orientations, distortions, and variations in object sizes and shapes, in this specific case, these random modifications during training only had negative effects. A possible explanation could be a mismatch with real-world data, as the augmentations might not accurately reflect the variability present in actual scenarios.

Lastly, the superior performance of YOLOv8 over the two-stage detector is consistent with findings from other recent studies (Butt et al., 2024). This could be attributed to the more recent advancements in the YOLO model, making it better equipped for our specific detection task.

### 4.1 Limitations

Despite the advancements in computer vision and deep learning techniques, insect detection remains a highly challenging task. One major obstacle is the limited availability of data essential for model training, necessitating the construction of our own insect dataset. Depending on the type of study, target objects can be exceedingly small, difficult to see, and may exhibit variability in terms of shape, color, wing poses, and decay conditions (Le et al., 2021), adding complexity to the creation of a robust and consistent dataset. Moreover, the acquisition process in an uncontrolled environment introduces various other forms of noise, including reflections, shadows, orientations, blurring, and variations in visual appearance.

As discussed in section 4, annotations in our study were not uniformly distributed across the dataset, particularly for the OI class, with labels concentrated in fewer densely populated images. Additionally, the condition of insects was often very compromised, potentially introducing noise and affecting model training. In this regard, we opted to label everything potentially related to the specific pests, despite the risk of increasing the number of false positives (such as misidentifying dry leaves as the ST class).

Finally, another factor to consider is the presence of other insects on the yellow traps, particularly other Cicadellidae species that closely resemble *S. titanus*, which may be erroneously identified by the model. Notable examples include *Fieberiella florii* and *Phlogotettix cyclops*, as highlighted in previous studies (Bosco et al., 1997; Chuche et al., 2010; Strauss and Reisenzein, 2018). To address this issue, our strategy involves the collection of digitized trap images. This simple yet efficient approach allows us to continuously enrich the dataset over time. By progressively incorporating more data, we can enhance the model’s capability to distinguish between highly similar species.

## 5 Conclusions

This study was initiated to evaluate the latest deep learning models for insect detection, aiming to take a significant step forward in the control of Flavescence dorée. The collected images constitute the first fully annotated dataset of *Scaphoideus titanus* and *Orientus ishidae*, which is now available to the scientific community (see Data availability section) and can be expanded over time focusing on a standardized and reproducible procedure. We trained deep learning models using YOLOv8 and Faster R-CNN architectures and conducted a benchmark analysis, providing valuable insights and operational tips for acquisition, augmentation and training processes. The two algorithms achieved mAP@0.5 scores of 0.92 and 0.86 respectively, demonstrating the effectiveness of object detectors in addressing this challenging problem. Moving forward, possible improvements could involve optimizing the acquisition process, enhancing image quality, and adding location details to track vector spread in vineyards. Additionally, a segmentation model could simplify field data acquisition by automatically cropping yellow traps before applying subsequent operations. The deployment of these models could establish an efficient monitoring network, opening up potential applications for field-use scenarios. Specifically, a smartphone tool capable of identifying FD vectors would not only enable farmers to take immediate action against the disease, but also allow the scientific community to continuously update the dataset, in the spirit of citizen science.

## Data Availability

The dataset presented in the study is publicly available at https://zenodo.org/records/11441757. For further details on the code, please refer to our GitHub repository, https://github.com/checolag/insect-detection-scripts.
