# Supplementary material for: A novel dataset and deep learning object detection benchmark for grapevine pest surveillance
Source: Front Plant Sci. 2024 Dec 12;15:1485216. doi: 10.3389/fpls.2024.1485216 (PMC11669504; doi:10.3389/fpls.2024.1485216)
Supplement: Supplementary file 1 [file DataSheet1.pdf]

Supplementary Material

1 Output YAML file of YOLOv8 models


```
task: detect
mode: train
model: yolov8s.pt
data: dataset.yaml
epochs: 300
time: null
patience: 100
batch: 16
imgsz: 1280
save: true
save_period: -1
cache: false
device: cuda:0
workers: 8
name: train
exist_ok: false
pretrained: true
optimizer: auto
verbose: true
seed: 0
deterministic: true
single_cls: false
rect: false
cos_lr: false
close_mosaic: 10
resume: false
amp: true
fraction: 1.0
profile: false
freeze: null
multi_scale: false
overlap_mask: true
mask_ratio: 4
dropout: 0.0
val: true
split: val
save_json: false
save_hybrid: false
conf: null
iou: 0.7
max_det: 300
half: false
dnn: false
plots: true
source: null
vid_stride: 1
stream_buffer: false
visualize: false
augment: false
agnostic_nms: false
classes: null
```

```
retina_masks: false
embed: null
show: false
save_frames: false
save_txt: false
save_conf: false
save_crop: false
show_labels: true
show_conf: true
show_boxes: true
line_width: null
format: torchscript
keras: false
optimize: false
int8: false
dynamic: false
simplify: false
opset: null
workspace: 4
nms: false
lr0: 0.01
lrf: 0.01
momentum: 0.937
weight_decay: 0.0005
warmup_epochs: 3.0
warmup_momentum: 0.8
warmup_bias_lr: 0.1
box: 7.5
cls: 0.5
dfl: 1.5
pose: 12.0
kobj: 1.0
label_smoothing: 0.0
nbs: 64
hsv_h: 0.015
hsv_s: 0.7
hsv_v: 0.4
degrees: 0.0
translate: 0.1
scale: 0.5
shear: 0.0
perspective: 0.0
flipud: 0.0
fliplr: 0.5
mosaic: 1.0
mixup: 0.0
copy_paste: 0.0
auto_augment: randaugment
erasing: 0.4
crop_fraction: 1.0
cfg: null
tracker: botsort.yaml
```

2 Output YAML file of Faster R-CNN models


```
CUDNN_BENCHMARK: false
DATALOADER:
  ASPECT_RATIO_GROUPING: true
  FILTER_EMPTY_ANNOTATIONS: false
  NUM_WORKERS: 8
  REPEAT_THRESHOLD: 0.0
  SAMPLER_TRAIN: TrainingSampler
DATASETS:
  PRECOMPUTED_PROPOSAL_TOPK_TEST: 1000
  PRECOMPUTED_PROPOSAL_TOPK_TRAIN: 2000
  PROPOSAL_FILES_TEST: []
  PROPOSAL_FILES_TRAIN: []
  TEST:
  - 1insect_val
  TRAIN:
  - 1insect_train
GLOBAL:
  HACK: 1.0
INPUT:
  CROP:
       ENABLED: false
       SIZE:
       - 0.9
       - 0.9
       TYPE: relative_range
  FORMAT: BGR
  MASK_FORMAT: polygon
  MAX_SIZE_TEST: 1333
  MAX_SIZE_TRAIN: 1333
  MIN_SIZE_TEST: 800
  MIN_SIZE_TRAIN:
  - 640
  - 672
  - 704
  - 736
  - 768
  - 800
  MIN_SIZE_TRAIN_SAMPLING: choice
  RANDOM_FLIP: none
MODEL:
  ANCHOR_GENERATOR:
       ANGLES:
       - - -90
       - 0
       - 90
       ASPECT_RATIOS:
       - - 0.5
       - 1.0
       - 2.0
       NAME: DefaultAnchorGenerator
       OFFSET: 0.0
       SIZES:
       - - 32
       - - 64
       - - 128
       - - 256
       - - 512
  BACKBONE:
       FREEZE_AT: 2
```

```
    NAME: build_resnet_fpn_backbone
  DEVICE: cuda
  FPN:
    FUSE_TYPE: sum
    IN_FEATURES:
    - res2
    - res3
    - res4
    - res5
    NORM: ''
    OUT_CHANNELS: 256
  KEYPOINT_ON: false
  LOAD_PROPOSALS: false
  MASK_ON: false
  META_ARCHITECTURE: GeneralizedRCNN
  PANOPTIC_FPN:
    COMBINE:
    ENABLED: true
    INSTANCES_CONFIDENCE_THRESH: 0.5
    OVERLAP_THRESH: 0.5
    STUFF_AREA_LIMIT: 4096
    INSTANCE_LOSS_WEIGHT: 1.0
  PIXEL_MEAN:
  - 103.53
  - 116.28
  - 123.675
  PIXEL_STD:
  - 1.0
  - 1.0
  - 1.0
  PROPOSAL_GENERATOR:
    MIN_SIZE: 0
    NAME: RPN
  RESNETS:
    DEFORM_MODULATED: false
    DEFORM_NUM_GROUPS: 1
    DEFORM_ON_PER_STAGE:
    - false
    - false
    - false
    - false
    DEPTH: 50
    NORM: FrozenBN
    NUM_GROUPS: 1
    OUT_FEATURES:
    - res2
    - res3
    - res4
    - res5
    RES2_OUT_CHANNELS: 256
    RES5_DILATION: 1
    STEM_OUT_CHANNELS: 64
    STRIDE_IN_1X1: true
    WIDTH_PER_GROUP: 64
  RETINANET:
    BBOX_REG_LOSS_TYPE: smooth_l1
    BBOX_REG_WEIGHTS: &id002
    - 1.0
    - 1.0
    - 1.0
```

```
    - 1.0
    FOCAL_LOSS_ALPHA: 0.25
    FOCAL_LOSS_GAMMA: 2.0
    IN_FEATURES:
    - p3
    - p4
    - p5
    - p6
    - p7
    IOU_LABELS:
    - 0
    - -1
    - 1
    IOU_THRESHOLDS:
    - 0.4
    - 0.5
    NMS_THRESH_TEST: 0.5
    NORM: ''
    NUM_CLASSES: 80
    NUM_CONVS: 4
    PRIOR_PROB: 0.01
    SCORE_THRESH_TEST: 0.05
    SMOOTH_L1_LOSS_BETA: 0.1
    TOPK_CANDIDATES_TEST: 1000
  ROI_BOX_CASCADE_HEAD:
    BBOX_REG_WEIGHTS:
    - &id001
    - 10.0
    - 10.0
    - 5.0
    - 5.0
    - - 20.0
    - 20.0
    - 10.0
    - 10.0
    - - 30.0
    - 30.0
    - 15.0
    - 15.0
    IOUS:
    - 0.5
    - 0.6
    - 0.7
  ROI_BOX_HEAD:
    BBOX_REG_LOSS_TYPE: smooth_l1
    BBOX_REG_LOSS_WEIGHT: 1.0
    BBOX_REG_WEIGHTS: *id001
    CLS_AGNOSTIC_BBOX_REG: false
    CONV_DIM: 256
    FC_DIM: 1024
    FED_LOSS_FREQ_WEIGHT_POWER: 0.5
    FED_LOSS_NUM_CLASSES: 50
    NAME: FastRCNNConvFCHead
    NORM: ''
    NUM_CONV: 0
    NUM_FC: 2
    POOLER_RESOLUTION: 7
    POOLER_SAMPLING_RATIO: 0
    POOLER_TYPE: ROIAlignV2
    SMOOTH_L1_BETA: 0.0
    TRAIN_ON_PRED_BOXES: false
    USE_FED_LOSS: false
```

```
    USE_SIGMOID_CE: false
  ROI_HEADS:
    BATCH_SIZE_PER_IMAGE: 256
    IN_FEATURES:
    - p2
    - p3
    - p4
    - p5
    IOU_LABELS:
    - 0
    - 1
    IOU_THRESHOLDS:
    - 0.5
    NAME: StandardROIHeads
    NMS_THRESH_TEST: 0.5
    NUM_CLASSES: 2
    POSITIVE_FRACTION: 0.25
    PROPOSAL_APPEND_GT: true
    SCORE_THRESH_TEST: 0.05
  ROI_KEYPOINT_HEAD:
    CONV_DIMS:
    - 512
    - 512
    - 512
    - 512
    - 512
    - 512
    - 512
    - 512
    LOSS_WEIGHT: 1.0
    MIN_KEYPOINTS_PER_IMAGE: 1
    NAME: KRCNNConvDeconvUpsampleHead
    NORMALIZE_LOSS_BY_VISIBLE_KEYPOINTS: true
    NUM_KEYPOINTS: 17
    POOLER_RESOLUTION: 14
    POOLER_SAMPLING_RATIO: 0
    POOLER_TYPE: ROIAlignV2
  ROI_MASK_HEAD:
    CLS_AGNOSTIC_MASK: false
    CONV_DIM: 256
    NAME: MaskRCNNConvUpsampleHead
    NORM: ''
    NUM_CONV: 4
    POOLER_RESOLUTION: 14
    POOLER_SAMPLING_RATIO: 0
    POOLER_TYPE: ROIAlignV2
  RPN:
    BATCH_SIZE_PER_IMAGE: 256
    BBOX_REG_LOSS_TYPE: smooth_l1
    BBOX_REG_LOSS_WEIGHT: 1.0
    BBOX_REG_WEIGHTS: *id002
    BOUNDARY_THRESH: -1
    CONV_DIMS:
    - -1
    HEAD_NAME: StandardRPNHead
    IN_FEATURES:
    - p2
    - p3
    - p4
    - p5
```

```
      - p6
      IOU_LABELS:
      - 0
      - -1
      - 1
      IOU_THRESHOLDS:
      - 0.3
      - 0.7
      LOSS_WEIGHT: 1.0
      NMS_THRESH: 0.7
      POSITIVE_FRACTION: 0.5
      POST_NMS_TOPK_TEST: 1000
      POST_NMS_TOPK_TRAIN: 1000
      PRE_NMS_TOPK_TEST: 1000
      PRE_NMS_TOPK_TRAIN: 2000
      SMOOTH_L1_BETA: 0.0
  SEM_SEG_HEAD:
      COMMON_STRIDE: 4
      CONVS_DIM: 128
      IGNORE_VALUE: 255
      IN_FEATURES:
      - p2
      - p3
      - p4
      - p5
      LOSS_WEIGHT: 1.0
      NAME: SemSegFPNHead
      NORM: GN
      NUM_CLASSES: 54
WEIGHTS:
https://dl.fbaipublicfiles.com/detectron2/COCO-Detection/faster_rcnn_R_50_FPN_3x/137849458
/model_final_280758.pkl
SEED: -1
SOLVER:
  AMP:
      ENABLED: false
  BASE_LR: 0.01
  BASE_LR_END: 0.0
  BIAS_LR_FACTOR: 1.0
  CHECKPOINT_PERIOD: 500
  CLIP_GRADIENTS:
      CLIP_TYPE: value
      CLIP_VALUE: 1.0
      ENABLED: false
      NORM_TYPE: 2.0
  GAMMA: 0.1
  IMS_PER_BATCH: 16
  LR_SCHEDULER_NAME: WarmupMultiStepLR
  MAX_ITER: 4000
  MOMENTUM: 0.9
  NESTEROV: false
  NUM_DECAYS: 3
  REFERENCE_WORLD_SIZE: 0
  RESCALE_INTERVAL: false
  STEPS: []
  WARMUP_FACTOR: 0.001
  WARMUP_ITERS: 1000
  WARMUP_METHOD: linear
  WEIGHT_DECAY: 0.0001
  WEIGHT_DECAY_BIAS: null
  WEIGHT_DECAY_NORM: 0.0
TEST:
```

```
  AUG:
       ENABLED: false
       FLIP: true
       MAX_SIZE: 4000
       MIN_SIZES:
       - 400
       - 500
       - 600
       - 700
       - 800
       - 900
       - 1000
       - 1100
       - 1200
  DETECTIONS_PER_IMAGE: 100
  EVAL_PERIOD: 100
  EXPECTED_RESULTS: []
  KEYPOINT_OKS_SIGMAS: []
  PRECISE_BN:
       ENABLED: false
       NUM_ITER: 200
VERSION: 2
VIS_PERIOD: 0
```

3 Practical tips

The Detectron2 library does not provide a validation loss during training by default. While `EVAL_PERIOD` setting allows to evaluate mean Average Precision (mAP) evolution, it is also essential to track the validation loss to prevent overfitting. To address this limitation, we developed a custom evaluator and added a new *hook* to the Trainer class (see the complete code on our GitHub repository): in Detectron2, a hook is a mechanism that allows you to register custom functions to be called at specific points during the training process. This hook calculates the loss in the same way it's done when training. During simulation runs, we observed that the selected two-stage model consumed a significant amount of GPU memory, exceeding 22 GB, while demanding minimal CPU RAM. Conversely, YOLOv8 required substantial GPU memory (around 16 GB) as well as CPU resources. Therefore, it's necessary to clear CUDA cache and delete model variables at the end of each training run.

4 Supplementary Figures and Tables

Supplementary Table 1. Comparison of augmentation hyperparameters between the default configuration and the training with no augmentation.

Test	hsv_h	hsv_s	hsv_v	degrees	translate	scale	shear	perspective	flipud	fliplr	mixup	copy_paste	erasing	crop_fraction	auto_agument
Default augmentation	0	0.7	0.4	0.0	0.1	0.5	0.0	0.0	0.5	1.0	0.0	0.0	0.4	1.0	randaugment
No augmentation	0	0	0	0	0	0	0	0	0	0	0	0	0	0	None

Supplementary Table 2. K-fold label distribution of the two insect classes: Scaphoideus titanus (ST) and Orientus ishidae (OI). The values represent the ratio of class instances between validation and training sets in each fold.

Folds	ST	OI
1	0.099	0.181
2	0.164	0.038
3	0.124	0.167
4	0.111	0.074
5	0.088	0.130
6	0.122	0.163
7	0.081	0.070
8	0.138	0.078
9	0.092	0.169
10	0.097	0.064

Supplementary Material

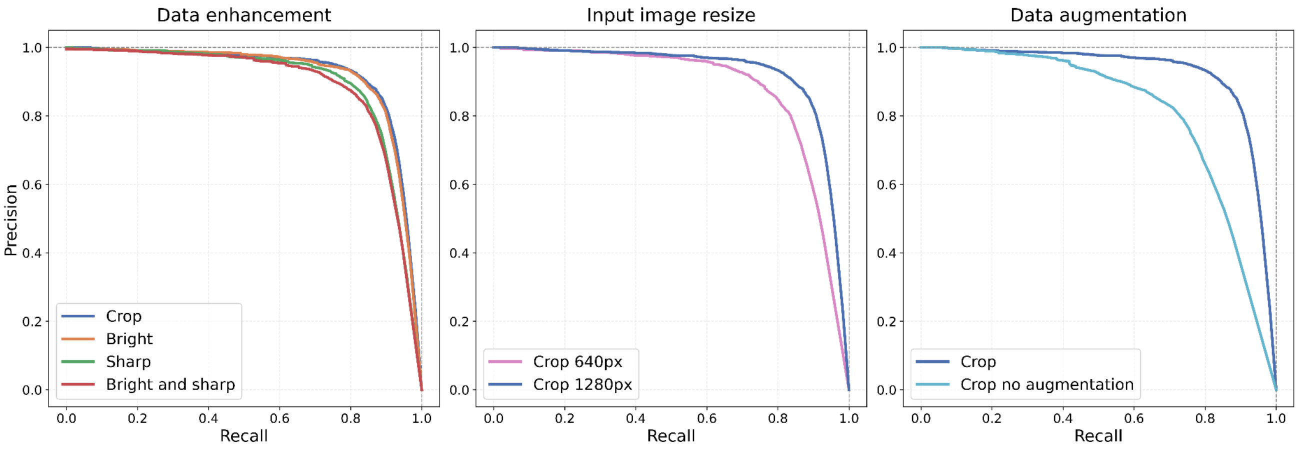

Supplementary Figure 1. Precision-Recall curves of experiments on input image modifications: from left to right the tests on data enhancements, input image size and data augmentation are examined.

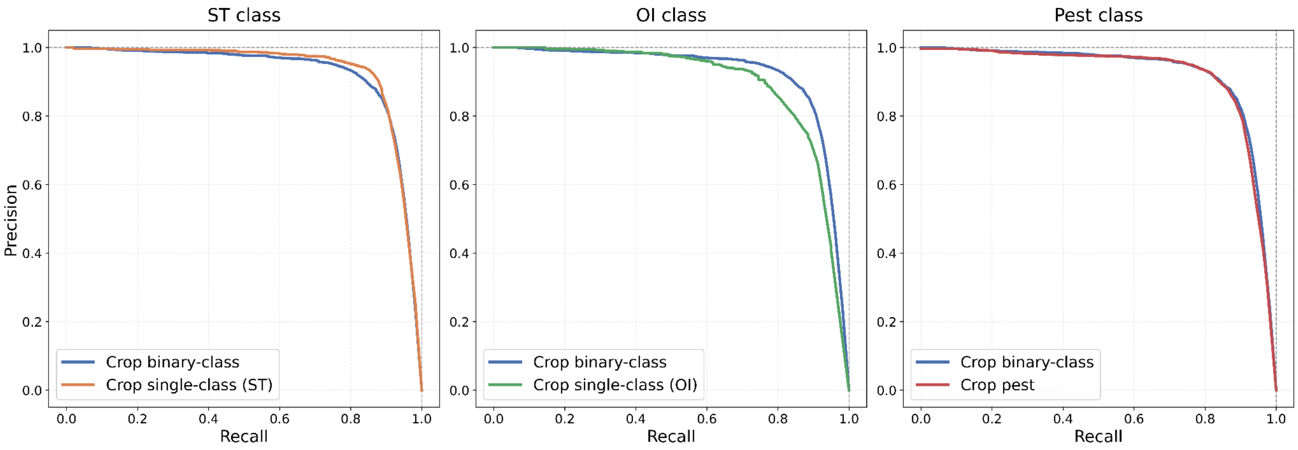

Supplementary Figure 2. Precision-Recall curves of class-oriented experiments: from left to right the focus is on Scaphoideus titanus (ST) class, Orientus ishidae (OI) class and single label 'pest'.

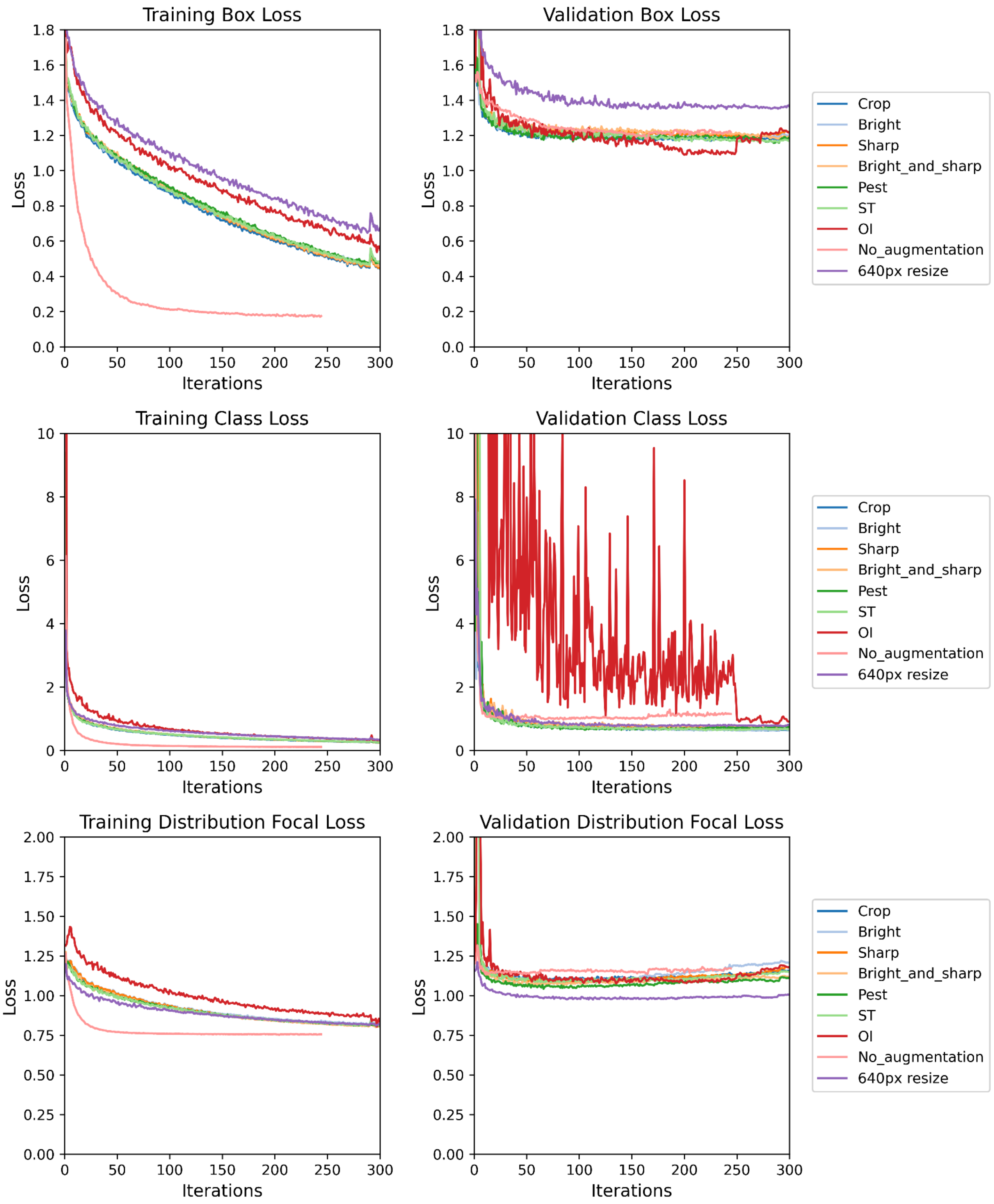

Supplementary Figure 3. Training and validation losses (Box, class and DFL) of YOLOv8 tests.

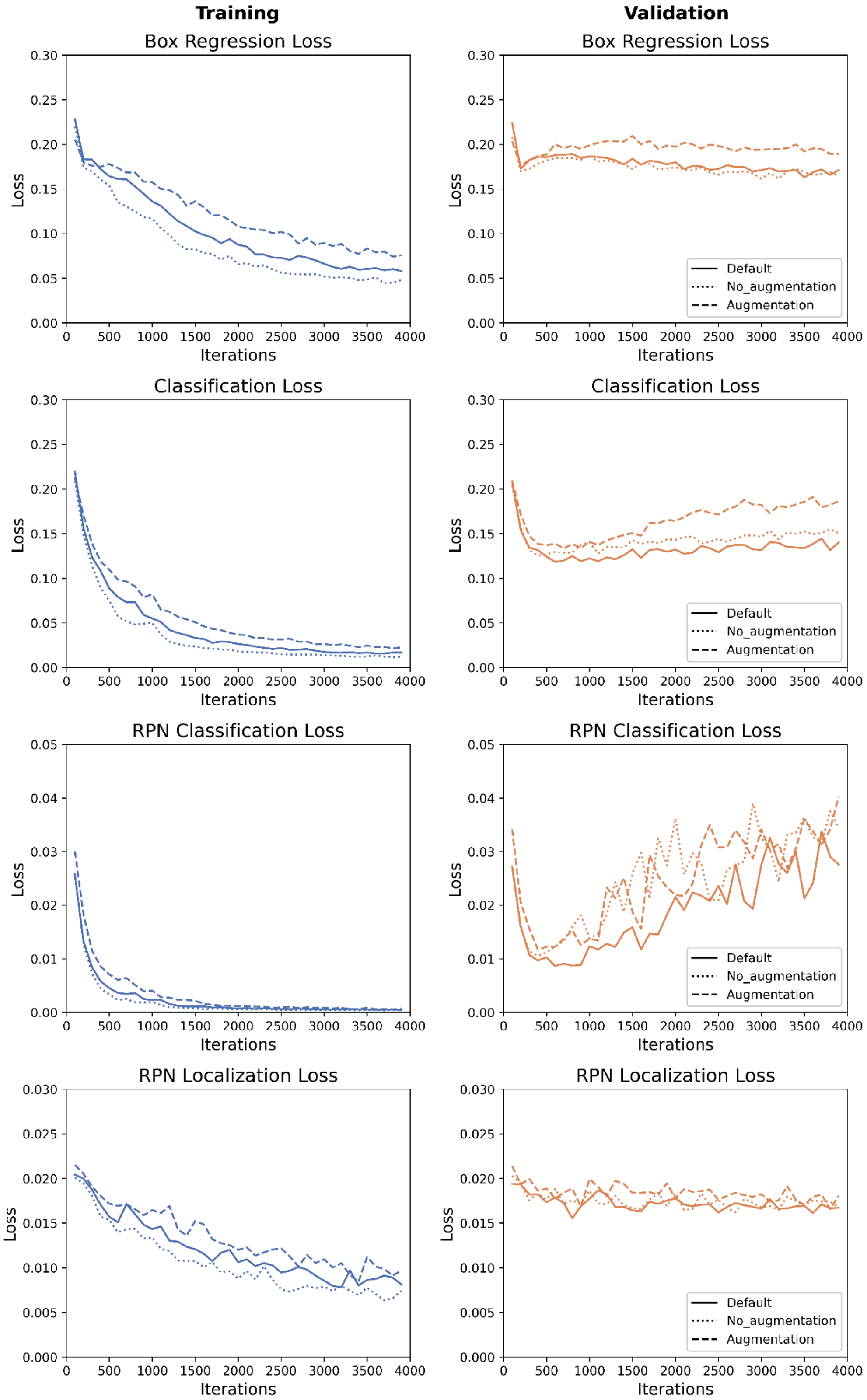

Supplementary Figure 4. Training and validation losses of Faster R-CNN tests.
